# Within-host genomic evolution of methicillin-resistant *Staphylococcus aureus* in long-term carriers

**DOI:** 10.1007/s00253-023-12932-3

**Published:** 2024-01-11

**Authors:** Tine Graakjær Larsen, Jose Alfredo Samaniego Castruita, Peder Worning, Henrik Westh, Mette Damkjær Bartels

**Affiliations:** 1 Department of Clinical Microbiology, Copenhagen University Hospital - Amager and Hvidovre, Copenhagen, Denmark; 2 Department of Infectious Disease Epidemiology and Prevention, Statens Serum Institut, Copenhagen, Denmark; 3 Department of Clinical Medicine, University of Copenhagen, Copenhagen, Denmark

**Keywords:** MRSA, Genomic evolution, cgMLST, SNP, Within-host, Recombination

## Abstract

**Abstract:**

Assessing the genomic evolution of *Staphylococcus aureus* can help us understand how the bacteria adapt to its environment. In this study, we aimed to assess the mutation rate within 144 methicillin-resistant *Staphylococcus aureus* (MRSA) carriers with a carriage time from 4 to 11 years, including some carriers who belonged to the same households. We found that 23 of the 144 individuals had completely different MRSA types over time and were therefore not long-term carriers of the same MRSA. From the remaining 121 individuals, we performed whole-genome sequencing (WGS) on 424 isolates and then compared these pairwise using core genome multilocus sequence typing (cgMLST) and single-nucleotide polymorphism (SNP) analyses. We found a median within-host mutation rate in long-term MRSA carriers of 4.9 (3.4–6.9) SNPs/genome/year and 2.7 (1.8–4.2) allelic differences/genome/year, when excluding presumed recombination. Furthermore, we stratified the cohort into subgroups and found no significant difference between the median mutation rate of members of households, individuals with presumed continued exposure, e.g., from travel and persons without known continued exposure. Finally, we found that SNPs occurred at random within the genes in our cohort.

**Key points:**

• *Median mutation rate within long-term MRSA carriers of 4.9 (3.4–6.9) SNPs/genome/year*

• *Similar median mutation rates in subgroups (households, travelers)*

• *No hotspots for SNPs within the genome*

**Supplementary Information:**

The online version contains supplementary material available at 10.1007/s00253-023-12932-3.

## Introduction

*Staphylococcus aureus* is a frequent human bacterial pathogen that can cause a wide range of infections, including invasive infections associated with high mortality (Tong et al. 2015). About 60% of humans are asymptomatic nasal carriers of *S. aureus* either permanently or intermittently (Kluytmans et al. 1997), and carriers of methicillin-resistant *S. aureus* (MRSA) are at risk of infections that can be difficult to treat because of few efficacious antibiotics (Datta and Huang 2008).

*S. aureus* has undergone extensive evolutionary adaptation to human and animal hosts and has the capacity to efficiently acquire and integrate antimicrobial resistance genes into its genome (Yebra et al. 2022). Many epidemic MRSA clones have been identified over time, with varying antimicrobial resistance and geographical prevalence (Turner et al. 2019). Some MRSA clones have been associated with epidemic spread in hospitals and some predominantly with livestock (Armand-Lefevre et al. 2005). Being able to perform genomic profiling of the MRSA strain involved in infection, colonization or causing outbreaks is an indispensable tool in the search to uncover how the bacteria evolves and adapts to changing environments (Fitzgerald and Holden 2016).

Studies have shown that *S. aureus* strains evolve with a rate of 1.2–3.3 × 10^−6^ single-nucleotide polymorphisms (SNPs) per site per year (Harris et al. 2010; Holden et al. 2013; McAdam et al. 2012; Nübel et al. 2013; Uhlemann et al. 2014), which in a genome of 2.85 million base pairs gives a rate of 3.4–9.4 SNPs/genome/year. Few studies have looked at the evolution of *S. aureus* in the same colonized individual (Azarian et al. 2019; Benoit et al. 2018; Golubchik et al. 2013; Goyal et al. 2019; Lagos et al. 2022). Goyal et al. (2019) included two persistent carriers of *S. aureus* (defined as *S. aureus* of same multilocus sequence type (MLST) at two different timepoints 3 years apart). The genomes of the isolates from each of the two carriers varied with a median of 20 SNPs (9–57 SNPs when pairwise SNP differences were calculated). A recent study from Sweden by Lagos et al. (2022) showed that among 20 long-term MRSA carriers, the median rate of allelic differences based on core genome MLST (cgMLST) was approximately 5/genome/year if the Ridom SeqSphere+ scheme was used (Ridom, Münster, Germany).

Danish National MRSA guidelines (Danish Health Authority 2016) recommend decolonization treatment of MRSA carriers and their household members. However, some MRSA carriers remain colonized even when systemic antibiotics are added to the decolonization regimen (Bagge et al. 2019). This study aims to describe the within-host evolution in 121 long-term MRSA carriers with a carriage duration of 4 to 11 years by analyzing genome data on 424 isolates. Furthermore, genomic data from long-term carriers within the same household will be assessed.

## Material and methods

### Study setting

The study was conducted at MRSA Knowledge Center, Department of Clinical Microbiology (DCM) at Hvidovre Hospital, Denmark. This DCM provides microbiological diagnostics for five large hospitals as well as approximately 700 general practitioners in the Southern and Central part of the Capital Region of Denmark, corresponding to an uptake area of about one million inhabitants. At DCM Hvidovre, 33 new MRSA cases were identified in 2003. This number increased each of the following years to 750 in 2019, which was approximately 20% of all new MRSA cases found in Denmark. Also in Denmark, there has been an increasing number of MRSA-positive patients over the last two decades as reported in the DANMAP report (DANMAP 2021), except in 2020 and 2021 ascribed to the restrictions imposed during the COVID-19 pandemic. MRSA from infection and carriage is notifiable in Denmark.

### Isolates and whole-genome sequencing database

*S. aureus* isolates suspected to be MRSA based on EUCAST cefoxitin disk diffusion breakpoints are confirmed to be MRSA using an in-house PCR with targets for the genes *nuc*, *fem*A, *mec*A, and *mec*C (Mollerup et al. 2022). A large part of the MRSA isolates comes from screening swabs from nose, throat, and perineum. These swabs are prior to cultivation on chromogenic MRSA plates and non-selective 5% horse blood agar inoculated into an MRSA tryptic soy broth containing 2.5% salt, 3.5 mg/L cefoxitin, and 20 mg/L aztreonam and incubated overnight.

Since 2003, typing has been performed on the first MRSA isolate from all patients, as well as on subsequent isolates from the same individual if more than a year has passed since the last typing or if the resistance pattern has changed. If a patient has more than one MRSA-positive sample on the same date, it is randomly chosen which isolate is whole-genome-sequenced. From 2003 to 2012, *spa* (staphylococcal protein A) typing by Sanger sequencing, detection of Panton Valentine leukocidin (PVL) genes and staphylococcal cassette chromosome *mec* (SCC*mec*) typing was used to assess genetic relatedness of MRSA isolates. Since 2013, whole-genome sequencing (WGS) of MRSA has been performed routinely in our laboratory.

As of January 2023, the local WGS database at DCM Hvidovre includes sequences from more than 10,000 MRSA isolates, mainly from the years 2013 to 2023, but also some earlier isolates that have been sequenced for various reasons.

### Inclusion criteria

The MRSA collection was assessed for isolates from patients that had been carriers for a period of at least 4 years. In cases where WGS data was available for all typed isolates of a patient, we compared the sequence types (STs) over time, and excluded patients if STs differed in at least three of the seven alleles during their carriage period (regarded as acquisition of a new clone). Patients who had a single outlier isolate at some time point during their 4 or more years of carriage were included if the 4 years of carriage was still applicable without the outlier, and only the outlier isolate was excluded. If a patient had isolates from before 2013 that had not been whole-genome-sequenced, we compared the *spa* types from the old isolates with the *spa* type and ST of the whole-genome-sequenced isolates. If the *spa* types were identical over time or if a single repeat was different or missing from one of the *spa* types but the remaining repeats were identical, indicating that it could still be the same clone, the old isolate was whole-genome-sequenced if it was necessary to obtain a 4-year time span. Patients that had very different *spa* types over time that were also known to typically belong to other STs than the one found in the whole-genome-sequenced isolates were excluded.

### Patient and sample information

The information included on patients and samples was available from the microbiology laboratory information system (LIS): Previous MRSA-positive samples, sampling dates for MRSA positive samples, sample site, clinical comments on travel information or contact to pig farms, and MRSA-positive household members. Age and gender information was determined from the Danish civil registration number. During analysis, all patients were anonymized by assigning a patient number (patient 1, 2, 3, etc.), and if they were part of a household with other long-term carriers included in this study, they were also given a household ID (household A, B, C, etc.).

### Whole-genome sequencing and bioinformatic analyses

From MRSA isolates, DNA was extracted using the DNeasy Blood and Tissue Kit (Qiagen, Hilden, Germany) from a single colony subcultured in serum broth (SSI Diagnostica, Hillerød, Denmark) and incubated for 24 h. Sequencing libraries were prepared using Nextera XT DNA sample preparation kit (Illumina, San Diego, CA, USA). The genomes were sequenced with 2×150 bp paired-end reads on an Illumina Miseq.

All raw read files were de novo assembled with SKESA (v.1.2) (Souvorov et al. 2018). The assembly quality was evaluated by having a total draft genome size of 2.6–3.1 Mbp, a minimum mean depth coverage of 30, and a minimum N50 of 10,000. Samples with low-quality assemblies were not included.

cgMLST was performed using SeqSphere+ v2.3-6.0 (Ridom, Münster, Germany) (Jünemann et al. 2013). The cgMLST scheme for *S. aureus* includes 1861 alleles. SeqSphere+ creates clusters for *S. aureus* with a threshold of 24 or fewer allelic differences and can create minimum-spanning trees (MSTs), distance matrices and neighbor-joining trees (NJTs).

Sequencing data were aligned, and SNPs were called using NASP pipeline (v.1.1.2) (Sahl et al. 2016). In the pipeline, duplicated regions in the reference were masked using nuccmer (v.3.1) (Delcher et al. 2003), reads were mapped to USA300_FPR3757 (NC_007793.1) reference genome with BWA-mem (v.0.7.16) (Li 2013), and SNPs were called with GATK (v.3.8.0) (Depristo et al. 2011; McKenna et al. 2010). Consensus bases had a minimum coverage of 10 and a minimum proportion of 0.9 for the called base. Pairwise comparisons between isolates of patients were performed using the consensus base matrix. Recombination regions were inferred using regions of elevated sequence diversity for every MLST group and filtered out using Gubbins (v.3.2.1) (Croucher et al. 2015). Supplemental Fig. S1 and Fig. S2 show the SNP rate per MLST group before and after using Gubbins. Bedtools (v.2.30.0) (Quinlan and Hall 2010) was used to find in which genes the intra-patient SNPs landed. Unless otherwise stated, all mentioning of SNPs in this text refers to SNPs after correction for recombination.

Lastly, the frequency of SNPs in 2809 genes was assessed using the reference genome USA300_FPR3757 (NC_007793.1) to determine whether specific genes in our cohort were mutated more often than others.

### Statistical analysis

All graphical presentations and statistical calculations were made using R (R Core Team 2022). Allelic differences in the 1861 core genes were assessed, as well as rate of SNP occurrence in the entire genome. An individual’s first isolate was considered their “day 0 isolate,” and mutation rate (SNPs/genome/year) was calculated by pairwise comparison of all secondary isolates with the individuals’ first isolate and dividing by the time since first sample. Medians and interquartile ranges were calculated for the cohort as whole, as well as for different subgroups by stratifying the cohort on different parameters. Mutation rates were compared using the Wilcoxon rank sum test; *p* values below 0.05 were considered statistically significant.

## Results

Of the initial 144 patients with isolates spanning 4 years or more, 23 were excluded because of likely reacquisition of a new clone. Thirteen were based on WGS data only, and all had STs that differed in five or more alleles over time, while 10 were based on comparisons of *spa* types of the old isolate and WGS of the newer isolates (e.g., a patient with t019 (usually ST30) and t026/ST45 or a patient with t044 (usually ST80) and t386/ST1). Six isolates from three different individuals were excluded as aberrant, but the individuals still fulfilled the inclusion criteria. The remaining cohort consisted of 121 individuals with 424 isolates. The cohort had been carriers for a period ranging from 4 to 11 years. The cohort included 63 females and 58 males. The median age at the time of their first MRSA sample was 40 years (range = 0–93 years). Seventy-five percent of the 424 isolates were screening samples, while the rest were clinical samples mainly from skin and soft tissue infections (77% of the clinical samples). Among the screening samples, 59% were throat samples, 28% were from nose, and 13% from perineum. Thirty-three of the patients (27 %) continuously had isolates from the same sample site sequenced repeatedly, while the rest of the patients had isolates from varying sample sites sequenced over time. As we did not have access to compare the data of the cohort with a control group (e.g., short-term carriers or MRSA negative persons), we were not able to identify patient factors related to long-term carriage.

### Cohort genomic characteristics

We used the pairwise comparison approach to compare as much of the genomes as possible as NASP will delete positions from the comparison that are not present in all isolates. Using the pairwise comparison approach, the average percentage of compared genome was 87.5% compared to only 50% if comparing all 424 isolates at the same time. Overall, 30 sequence types (MLST), 57 *spa*-types, and 133 cgMLST complex types (CT) were represented (see online Fig.: https://microreact.org/project/5ceChMtMXMqSEoTifbeCLo-mrsa-long-term-carriage). ST6, ST22, and ST8 constituted more than a quarter of all isolates.

### Genomic evolution over time

**Fig. 1:**
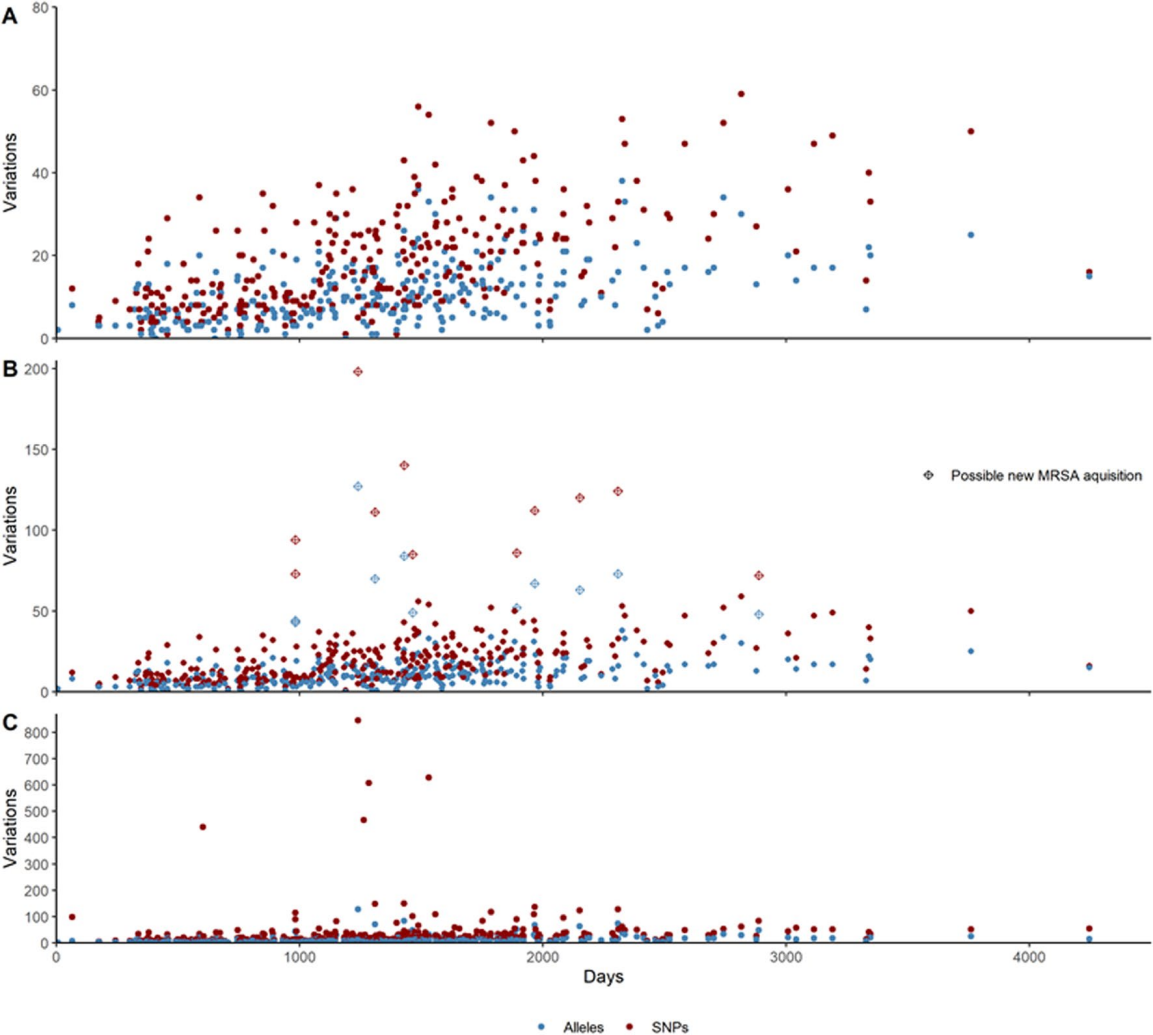
Scatterplot showing the number of SNPs (red) and allelic differences (blue) for all secondary isolates from an individual when performing pairwise comparison to the individual’s first isolate. The x-axis is days from first isolate, y-axis is number of SNPs/allelic differences. **A** The final cohort of included isolates after correcting for recombination and exclusion of possible new acquisitions. **B** After correcting for recombination. Eleven possible new MRSA acquisitions are shown with a different symbol. **C** Variations over time in all individuals before correction for recombination

The number of SNPs and allelic differences occurring over time is illustrated in Fig. 1. The overall SNP rates decreased from a median of 5.8 (3.8–13.0) SNPs/genome/year to 4.9 (3.4–6.9) SNPs/genome/year after correcting for recombination while the allelic differences/genome/year remained 2.7 (1.8–4.2). The correction for recombination filtered out regions related to mobile genetic elements and phage related genes.

After correction for recombination, there were still 11 isolates with more than 70 SNPs from the first isolate; these are marked in Fig. 1B. These isolates belonged to eight different individuals, none of them living in households with other long-term carriers. Seven out of eight of these individuals had information in the LIS that indicated possible continuous exposure: either from frequent travel to MRSA endemic areas or work with pigs, making it possible that they had acquired a new strain with a similar genetic background, rather than the evolution having taken place solely on them. If these 11 isolates were excluded from the dataset, the overall mutation rate within the cohort would remain the same, 4.9 (3.4–7.3) SNPs/genome/year and 2.6 (1.8–4.2) allelic differences/genome/year.

### ST 398

Eight individuals within the cohort were found to have the livestock associated MRSA ST 398. All but one of the eight individuals had information about working with live pigs. In Fig. 1A, four of the ST 398 isolates from three different persons had more than 400 SNPs compared to their first isolates, but after correcting for recombination, the number of SNPs for three of these isolates were reduced to a normal number of SNPs indicating that they did carry the same strain over time. The number of SNPs for the last person was reduced from 845 to 198 and was therefore a probable new acquisition (Fig. 1B).

### Travel and other continuous exposure sources

**Fig. 2:**
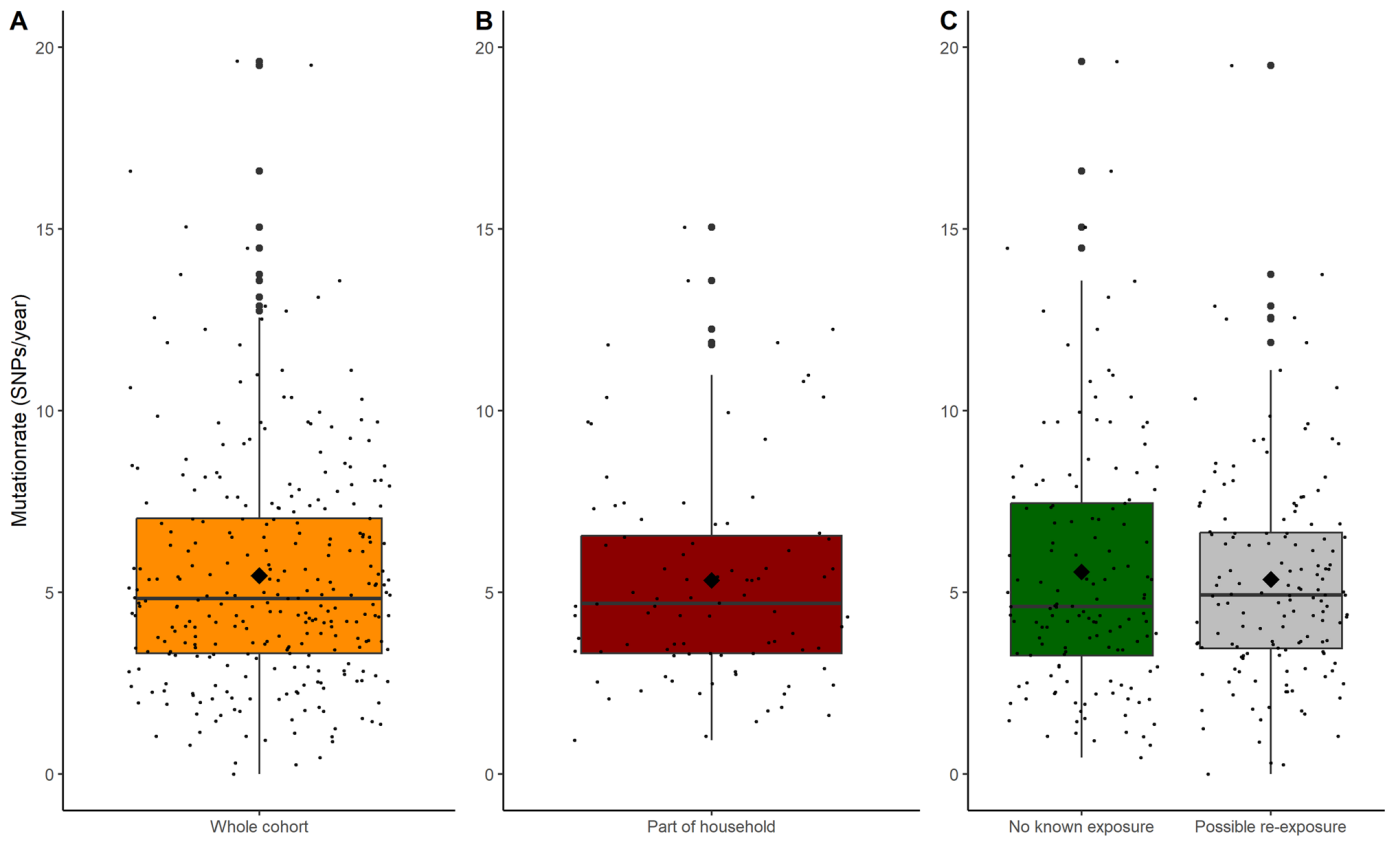
Mutation rate (SNPs after correcting for recombination) shown as boxplots on top of jitterplots. The diamond shape shows the mean. **A** includes the entire cohort. **B** includes isolates from individuals belonging to households with other long-term MRSA carriers within the cohort. **C** Comparison of the group of individuals with known possible re-exposure to MRSA and the group on whom no known continuous exposure is known

According to travel information in the LIS, almost half (*n*=55) of the cohort was identified as “possible frequent travelers to countries with a high prevalence of MRSA” (Lee et al. 2018). Other forms of continuous exposure were identified in the LIS, e.g., intravenous drug use, homelessness, and working with live pigs. Within the cohort, 55 individuals either did not have any of known exposures, or information on this was lacking from the LIS. The median mutation rate in this group without any identified exposures was 4.6 (3.3–7.7) SNPs/genome/year and 2.6 (1.6–4.3) allelic differences/genome/year. This was lower than the median mutation within the group of individuals with possible continuous exposure, but not significantly (*p* = 0.4) (see Fig. 2).

### Households

**Fig. 3:**
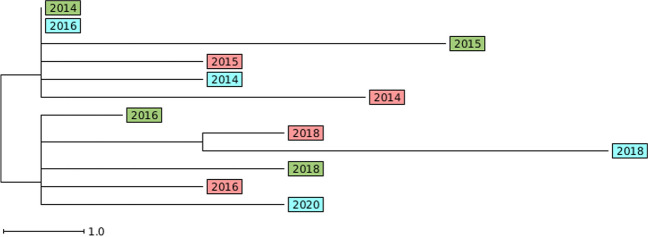
Neighbor-joining tree based on output from SeqSphere+ (cgMLST) of a household with three long-term carriers. Each color represents an individual in the household and the year within the circle is the year the sample was taken. The scale bar corresponds to the number of cgMLST allelic differences

Within the cohort there were 36 individuals who came from 16 households with other long-term carriers: siblings, parent-child, husband-wife, etc. The mutation rate within the group of individuals who belonged to households (130 isolates in total) was estimated to be 4.8 (3.3–6.8) SNPs/genome/year and 2.6 (1.6–3.9) allelic differences/genome/year. This was similar to the cohort as a whole (see Fig. 2). Figure 3 shows an example of a neighbor-joining tree (NJT) of a household with three long-term carriers with four isolates each (ST 1457, *spa* t069) over a period of 6 years. The household members were swabbed for the first time within 4 months of each other. The figure shows that in some cases the temporally associated isolates are more genetically related than isolates from the same individual.

### Locating hotspots for mutations in the genome

We searched for the frequency of SNPs in 2809 genes using the reference genome USA300_FPR3757 (NC_007793.1) to determine whether some genes were more often mutated than others in our cohort. We found no SNPs in 1255 of the genes in any individual within the cohort. The gene where most individuals had SNPs (*n* = 52) was the *ebh* gene which codes for an extracellular matrix binding protein. However, *ebh* is by far the largest gene, with a base pair length of 31,267. Normalizing the number of cases by the gene length the *nirD* gene, which is within the nitrate reductase *nir* cluster, was the most commonly mutated gene with eight individuals having SNPs within the gene’s 315 basepairs.

## Discussion

The study aimed to assess the within-host and within-household genetic evolution of MRSA by including 424 isolates from 121 long-term MRSA carriers each colonized with the same strain for 4 to 11 years. The cohort was diverse regarding MLST, cgMLST and *spa* types. We found that 16% (23 out of the initial 144 carriers) of presumed long-term carriers were in fact recolonized with new MRSA strains of completely different genetic backgrounds. In addition, seven patients probably had reacquisitions of MRSA with similar genetic backgrounds over time likely due to traveling to MRSA endemic areas where their clones are common. This finding gives a new insight into long-term carriage of MRSA as it shows that intermittent carriers can be misinterpreted as permanent carriers when detailed typing data is not available. Furthermore, if these patients have undergone decolonization treatment, positive control swabs might be interpreted as treatment failure, thereby underestimating the effect of the treatment.

We estimated an overall median mutation rate for the entire cohort of to 4.9 (3.4–6.9) SNPs/genome/year and 2.7 (1.8–4.2) allelic differences/genome/year. This finding is similar to previously reported estimates of *S. aureus* mutation rates (Azarian et al. 2019; Benoit et al. 2018; Golubchik et al. 2013; Goyal et al. 2019; Lagos et al. 2022; Young et al. 2012). Azarian et al. (2019) analyzed *S. aureus* isolates from four cystic fibrosis patients over a period of approximately four years and found a mutation rate of 2.8-5.5 SNPs/genome/year. Lagos et al. (2022) evaluated the evolution of MRSA within 95 isolates from 20 long-term carriers, including one that had carried the strain for 11 years and estimated a genomic variation rate of 2.0–5.8 genetic events per year. The study also used SeqSphere+ (among other schemes) for assessment of WGS results. Both studies found comparable mutation rates to ours. The methodologies used to assess mutation rates, however, can differ between studies. While the current study aimed to describe the mutation rates by calculating medians of pairwise comparison, other studies may use slightly different approaches. Also, as Lagos et al. (2022) stated in their paper, the observed mutation rate is highly dependent on the scheme used to assess WGS variations. Keeping this in mind, drawing conclusions based on comparisons of estimates from different studies should be done with care.

Due to the large cohort size, we were able to stratify according to some known exposures that could increase the risk of new acquisition and thereby affect the calculated mutation rate independently of within-host evolution. We stratified the cohort based on whether there was information about frequent travel to MRSA endemic areas in the LIS. It is well-established that individuals who frequently travel to visit friends and relatives in other countries are at risk of becoming infected or colonized with microorganisms prevalent in these areas (Leder et al. 2006). We found that isolates from carriers with frequent travel to MRSA endemic countries had a slightly higher, yet comparable, median SNP rate/genome/year than non-frequent travelers (4.9 SNPs versus 4.6 SNPs). Some of the “frequent travelers” may have been recolonized upon travel, with a strain of identical *spa* and MLST type, but which had undergone evolution since the last encounter with it. Hence, the observed evolution had not taken place within the same host. However, a limitation to this study is, that we do not have complete travel and exposure information on all patients and the grouping of patients into frequent travelers and non-frequent travelers is therefore deficient. In addition, several other factors that could increase the risk of new acquisitions and thereby influence on the mutation rates are not included in our dataset.

Another exposure increasing the risk of acquisition of new clones is daily contact to pigs. The cohort included eight persons with the livestock associated MRSA ST 398 of which seven where known to have contact to live pigs. In the first SNP analysis, four isolates from three of these persons had a very high SNP rate, and our thought was that it could be due to acquisition of different strains from the pig farm. However, after correcting for recombination, the SNP rate was reduced to a level comparable to all other isolates for three of the isolates, while the fourth isolate was unaffected, thus indicative of being a new acquisition. Correcting for recombination is important when estimating the SNP rate. In our study, the SNP rate was reduced from 5.8 (3.8–13.0) SNPs/genome/year to 4.9 (3.4–6.9) after correction for recombination, while the number of allelic differences were not affected, because the recombinant regions did not affect the core genes. A low rate of recombination in the core genes and a high rate of recombination in the non-core genes has previously been reported (Castillo-Ramírez et al. 2011), and the importance of considering recombination when assessing mutation rates has been emphasized by others (Everitt et al. 2014; Lagos et al. 2022).

We identified 16 households within the cohort. The evolution observed in the individual household members did not appear to occur solely within themselves, but rather as a collective change affecting the entire household. It is likely that back-and-forth transmission among household members had been taking place which has also been suggested by Uhlemann et al. (2014). Household longitudinal *S. aureus* transmission dynamics have previously been investigated, including transmission among household members, from fomites and from pets (Mork et al. 2020). Mork et al. (2020) pointed out that, in addition to physical contact and a high burden of colonization among household members, environmental contamination seems to play a key role in the transmission within households. We found that the group of individuals belonging to households with other long-term carriers had a similar yet slightly lower median mutation rate than individuals not living with other long-term carriers (4.8 (3.3–6.8) SNPs/genome/year compared to 5.0 (3.5–7.9) SNPs/genome/year). In two USA300 studies including isolates from households, mutation rates were estimated to be approximately 3 to 3.5 SNPs/genome/year (Alam et al. 2015; Uhlemann et al. 2014), which is lower than our finding.

SNPs within the cohort did not show a pattern within certain genes. Instead, mutations appeared to occur at random, with longer genes not surprisingly being more likely to have SNPs.

A limitation to this study is that only one isolate was analyzed per sample. Several studies have shown that a “cloud of diversity” exists within a carrier and that individual colonies from the same sample can differ by up to 40 SNPs (Golubchik et al. 2013; Goyal et al. 2019; Paterson et al. 2015). Although our quality control settings reduced the risk of sequencing mistakes, it cannot be ruled out that less or more mutations were identified than were actually present in the genomes of our cohort. In this study, there was an observable trend in the occurrence of mutations over time, thus suggesting that selecting only one isolate from each sample still provides useful information about genomes of colonizing MRSA and the evolution that occurs within them. Another possible challenge to our results is that the sample site of the whole-genome-sequenced isolates differed for 73% of patients; e.g., in some cases, the “day 0” isolate was from an infection, and the secondary isolates were follow-up pharyngeal screening samples. It is well known that the milieu of different anatomic locations can differ regarding factors such as pH, humidity, and nutrient availability and thereby affect the adaptation and gene expression of *S. aureus* (Dastgheyb and Otto 2015)*.* Giulieri et al. (2022) studied an international collection of 2590 *S. aureus* genomes from both colonization and infection and found that a small number of genes often acquired mutations during infection. On the other hand, Hall et al. (2019) analyzed genomic data from a hospital outbreak where 27 patients had samples from at least two time points or from two body sites or both but did not find indications of phylogeny/body site correlation. Some of the long-term carriers in our study had both clinical and screening isolates, and it is possible that the mutation rate was affected by this compared to cases where only isolates from the same site were whole-genome-sequenced.

To our knowledge, this is the largest and longest study of within-host evolution in MRSA carriers to date. After correcting for recombination, we found a median within-host evolution in long-term MRSA carriers of 4.9 (3.4–6.9) SNPs/genome/year or 2.7 (1.8–4.2) allelic differences/genome/year. When stratification was done, mutation rate within subgroups was similar to the overall mutation rate, although the median mutation rate tended to be lower in individuals belonging to households with other carriers and higher among individuals with repeated exposures like frequent travel to high endemic areas. We found that SNPs occurred at random within the genes in our cohort. Although only one colony per sample was whole-genome-sequenced and thus a possible cloud of diversity in each patient was not obtained, this approach seems acceptable to estimate the within-host evolution in long-term MRSA carriers.

## Supplementary information

ESM 1 (PDF 145 kb)

## Data Availability

The WGS data analyzed in the current study are available at https://www.ncbi.nlm.nih.gov/bioproject in the following bioprojects: PRJNA991754 (327 samples), PRJNA691722 (13 samples), PRJNA704240 (2 samples), PRJNA839593 (70 samples), PRJNA865897 (3 samples), PRJNA869909 (7 samples), PRJNA893597 (1 sample), and PRJNA898141 (1 sample).
