# Supplementary material for: Within-host genomic evolution of methicillin-resistant Staphylococcus aureus in long-term carriers
Source: Appl Microbiol Biotechnol. 2024 Jan 11;108:95. doi: 10.1007/s00253-023-12932-3 (PMC10784349; doi:10.1007/s00253-023-12932-3)
Supplement: Supplementary file 1 [file 253_2023_12932_MOESM1_ESM.pdf]

Supplemental Information

Applied Microbiology and Biotechnology

Within-host genomic evolution of methicillin-resistant *Staphylococcus aureus* in long-term carriers

Tine Graakjær Larsen[1,2], Jose Alfredo Samaniego Castruita[1], Peder Worning[1], Henrik Westh[1,3], Mette Damkjær Bartels[1,3]

1. Department of Clinical Microbiology, Copenhagen University Hospital - Amager and Hvidovre, Copenhagen, Denmark.

2. Department of Infectious Disease Epidemiology and Prevention, Statens Serum Institut, Copenhagen, Denmark

3. Department of Clinical Medicine, University of Copenhagen, Copenhagen, Denmark

Corresponding author:

Mette Damkjær Bartels

e-mail: mette.damkjaer.bartels@regionh.dk

Phone +45 38626503 Fax +45 38623357

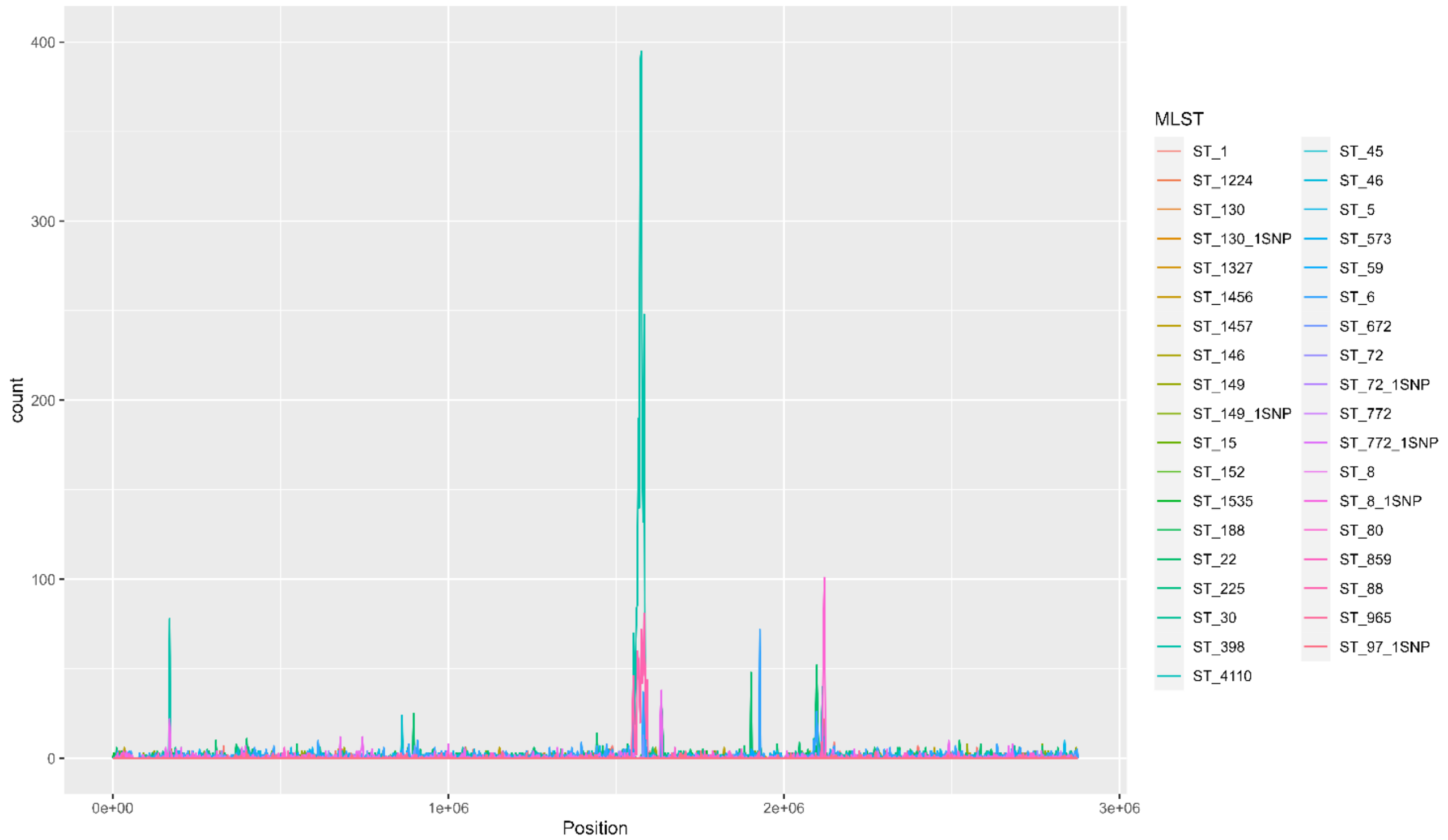

Figure S1

The plot shows the number of SNPs across the genome grouped by MLST before removing "recombination regions" using Gubbins. The highest peak represents isolates belonging to ST398.

X-axis: Position in the genome. Y-axis: Number of SNPs

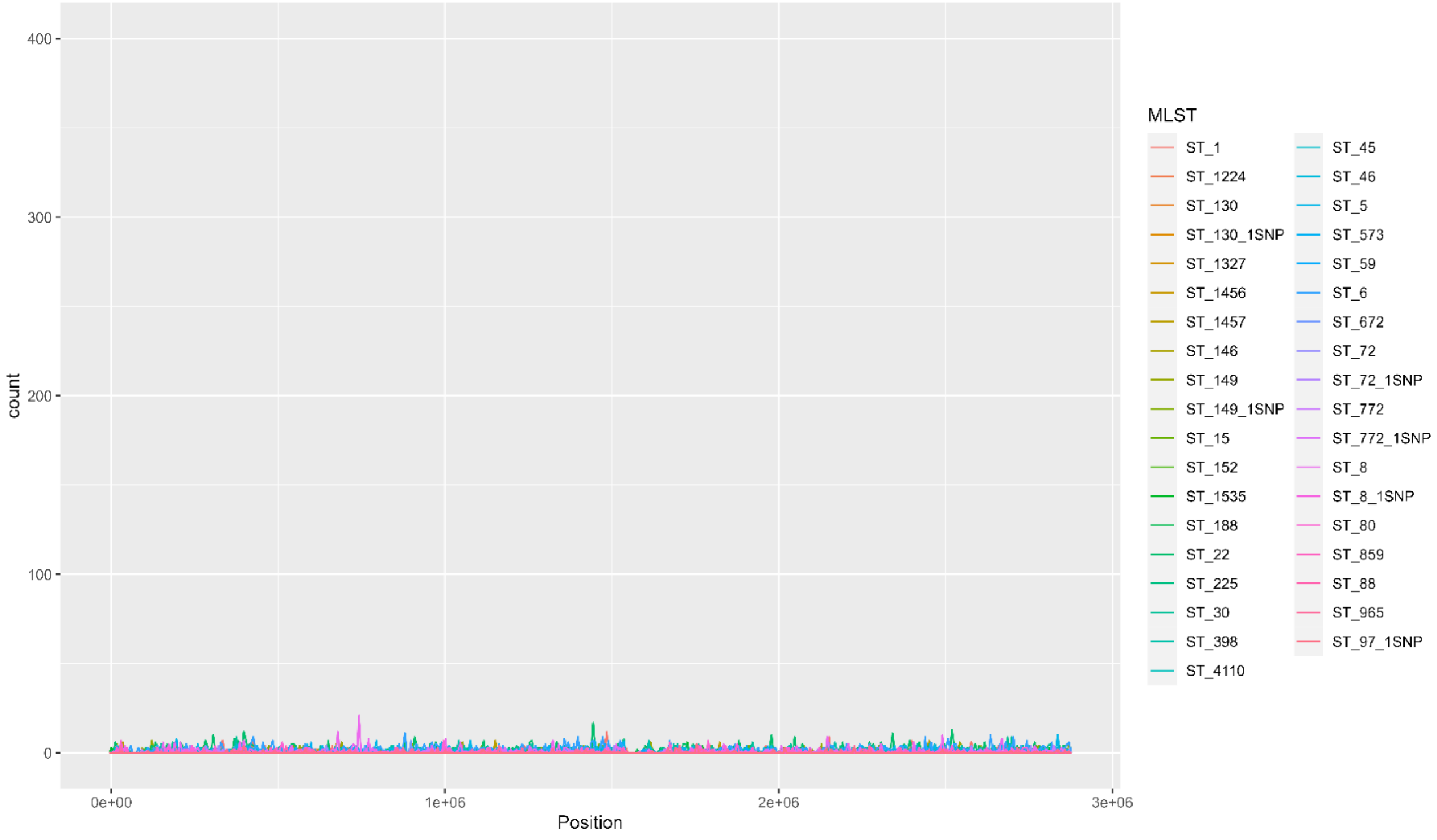

Figure S2

The plot shows the number of SNPs across the genome grouped by MLST after removing "recombination regions" using Gubbins. The high peaks seen in Figure S1 have disappeared. X-axis: Position in the genome. Y-axis: Number of SNPs
